# The Etiology of Kidney Failure in Indonesia: A Multicenter Study in Tertiary-Care Centers in Jakarta

**DOI:** 10.5334/aogh.4071

**Published:** 2023-06-01

**Authors:** Ni Made Hustrini, Endang Susalit, Aida Lydia, Maruhum Bonar H. Marbun, Muhammad Syafiq, Yassir, Johanes Sarwono, Elizabeth Yasmine Wardoyo, Jonny, Suhardjono, Rizki Y. Pradwipa, Anitasari Nugraheni, Merel van Diepen, Joris I. Rotmans

**Affiliations:** 1 Division of Nephrology and Hypertension – Department of Internal Medicine, Faculty of Medicine – Universitas Indonesia, Dr. Cipto Mangunkusumo National General Hospital, Jakarta, ID; 2 Department of Internal Medicine, Leiden University Medical Center, Leiden, NL; 3 Department of Internal Medicine, Persahabatan General Hospital, Jakarta, ID; 4 Division of Nephrology – Department of Internal Medicine, Fatmawati General Hospital, Jakarta, ID; 5 Division of Nephrology, Department of Internal Medicine, Gatot Soebroto Central Army Hospital, Jakarta, ID; 6 Hemodialysis Unit, Pelni General Hospital, Jakarta, ID; 7 Department of Clinical Epidemiology, Leiden University Medical Center, Leiden, NL

**Keywords:** etiology of kidney failure, primary renal disease, dialysis, kidney transplantation, multicenter

## Abstract

**Background:**

Despite a large number of patients requiring dialysis, the etiology of kidney failure is poorly documented in Indonesia. With the aim to reduce the disease burden, it is essential to obtain more insight in the etiology of chronic kidney disease (CKD).

**Objective(s):**

In the present study, we attempted to investigate the primary renal disease of kidney failure patients from five tertiary-care centers in Jakarta.

**Methods:**

This is a multicenter, cross-sectional study of kidney failure patients receiving kidney replacement therapy (KRT), from December 2021 to July 2022. We recruited patients aged ≥18 years, had been receiving dialysis for at least three months or a kidney transplantation.

**Findings:**

This study included 1,152 patients treated with hemodialysis (68.1%), peritoneal dialysis (7.5%), and kidney transplantation (24.4%). At the start of KRT, the median (interquartile-range [IQR]) age was 48 [37–58] years with low eGFR (median [IQR]: 5.9 [4.0–8.34] ml/minute/1.73 m^2^). Hypertension was the main comorbidity (74.2%), followed by diabetes mellitus (30.1%). The major primary kidney disease was diabetic kidney disease (27.2%), followed by glomerulonephritis (13.0%), hypertension (11.5%), and urolithiasis (10.3%). Lupus nephritis was the common underlying etiology of secondary glomerulonephritis (91%). A high rate of unknown cause (31.1%) was also observed.

**Conclusions:**

Our results suggest that diabetic kidney disease is the leading cause of kidney failure in Jakarta, followed by glomerulonephritis. This study highlights the need for a better approach on primary prevention of diabetes mellitus as well as to better recognize glomerulonephritis at earlier stage might have a significant impact on reduction of the rate of kidney failure in Indonesia.

## Introduction

The chronic kidney disease (CKD) burden in Indonesia continues to rise. Based on the Centers for Disease Control and Prevention (CDC), both kidney diseases and diabetes mellitus (DM) are the third leading cause of death in Indonesia [1]. Although the prevalence of CKD remains elusive, the number of patients undergoing kidney replacement therapy (KRT), largely in the form of hemodialysis, is steadily increasing. Since the National Health Coverage was initiated in 2014, the prevalence of chronic hemodialysis patients has risen by 84% in five years, reaching to 132,000 in 2018 [2]. Consequently, the costs associated with chronic hemodialysis treatment have also increased, making kidney failure the fourth leading cause of health expenditure after heart disease, cancer and stroke [3].

Despite high number of patients receiving dialysis, the etiology of kidney failure is not well documented in Indonesia. Most of the patients who need dialysis have an urgent start, limiting clinical efforts to identify the cause of kidney failure [4]. This suggests a lack of awareness of individual kidney health, the need for effective screening and prevention programs for high-risk populations, and a referral system for noncommunicable diseases (NCDs). To design such program, there is an urgent need to map the magnitude of CKD, including CKD-related outcomes, progression to kidney failure as well as the financial burden of KRT.

Multiple risk factors are known to be associated with CKD development. Currently, Indonesia is in epidemiological transition period in which NCDs are progressively critical, while infectious diseases continue to be a substantial part of the disease burden. The National Basic Health Survey (2007–2018) showed a progressive rise in metabolic diseases related to CKD progression [5,6,7,8]. Additionally, our study revealed a high prevalence of hypertension among adults over 18 years of age, reaching 41% [9]. Furthermore, the high prevalence of communicable diseases e.g., malaria, tuberculosis, diarrhea, or dengue, similarly affect CKD prevalence through associated episodes of acute kidney injury. Besides, multiple other factors could influence CKD development in Indonesia, include high-salt intake, a sedentary lifestyle, exposure to nephrotoxic agents, inadequate access to clean water, high temperatures, and limited access to health care facilities. These facts may demonstrate how Indonesia differs from western countries, where the majority of available data have been collected.

In the present study, we aim to investigate the etiology of kidney failure patients in five tertiary-care hospitals in Jakarta by 1) describing its distribution; 2) stratifying by gender, age, and KRT modalities; and 3) describing kidney function related to different primary renal diseases (PRD).

## Methods

### Study design and participants

This is a multicenter, cross-sectional study of kidney failure patients receiving KRT (i.e., dialysis or kidney transplantation) from December 2021 to July 2022 in Jakarta. The study was conducted at five major hospitals, including Dr. Cipto Mangunkusumo National General Hospital, Persahabatan General Hospital, Fatmawati General Hospital, Gatot Soebroto Army Central Hospital, Pelni General Hospital.

Inclusion criteria including: 1) aged ≥18 years; 2) have undergone hemodialysis or peritoneal dialysis for at least three months; or have received a kidney transplantation. The exclusion criteria are as follows: 1) patients who have encountered acute kidney injury and are temporarily dependent on dialysis; or 2) those who are unable to communicate.

Written informed consent was obtained from all participants, and this study had received ethical approval from The Ethic Committee of the Faculty of Medicine, Universitas Indonesia – Dr. Cipto Mangunkusumo Hospital, number: KET-1065/UN2.F1/ETIK/PPM.00.02/2021 by 1 November 2021.

The clinical and research activities being reported are consistent with the Principles of the Declaration of Istanbul as outlined in the “Declaration of Istanbul on Organ Trafficking and Transplant Tourism” as well as to the “Declaration of Helsinki” on the ethical principles regarding human experimentation.

### Variables and measurement

Each subject underwent a structured interview to gather information on their family history of kidney disease, smoking habit, history of kidney stone, and exposure to nephrotoxic substances such as such as non-steroid anti-inflammatory drugs (NSAIDs), traditional/alternative medications, carbonated drinks (soda, taurine), alcohol.

The subjects’ hemodialysis vascular access was examined and categorized as either arteriovenous fistula, arteriovenous graft, or central vein catheter.

Demographic data, kidney failure etiology as diagnosed by a nephrologist or treating physician, date of initiation of KRT, dry weight, body height, blood pressure, comorbidities, and medications (including angiotensin-II receptor blockers (ARBs)/angiotensin converting enzyme inhibitors (ACE-i), phosphate binders, statins, erythropoiesis stimulating agents, and immunosuppressants) were reviewed from the subjects’ medical records.

Laboratory parameters at the presentation of kidney disease or at the start of KRT, such as hemoglobin, serum albumin, serum calcium, serum phosphate, serum sodium, serum potassium, estimated glomerular filtration rate (eGFR), hepatitis B surface antigen (HBsAg), antibody hepatitis C virus (Anti-HCV), and antibody human immunodeficiency virus (Anti-HIV), as well as serological/immunological data needed to establish the etiology of kidney disease (performed at the presentation of kidney disease or at the start of KRT) such as anti-nuclear antibody (ANA), anti-double stranded DNA (anti-dsDNA), complement-3 (C3), complement-4 (C4), immunoglobulin A (IgA), anti-cardiolipin immunoglobulin-G (ACA IgG), anti-cardiolipin immunoglobulin-M (ACA IgM), beta-2 glycoprotein immunoglobulin-G (Beta-2 GP IgG), beta-2 glycoprotein immunoglobulin-M (Beta-2 GP IgM), urinalysis, urine albumin (or protein) to creatinine ratio, 24-hour quantitative proteinuria, imaging (kidney ultrasound, kidney CT-scan, kidney MRI, abdominal x-ray, chest x-ray), and echocardiography were also recorded. Histopathological data from kidney biopsy was also extracted from the subject’s medical record.

Estimated glomerular filtration rate (eGFR, ml/min/1.73 m^2^) data was extracted from medical record at the KRT commencement and the formula used to calculate the eGFR were the MDRD formula and CKD-EPI equation.

Diagnosis of hematuria was based on microscopic data (urinalysis examination). It was presented as the number of cells per high power field (i.e., ≥5 red blood cells/HPF). Likewise, the diagnosis of proteinuria was based on the urine albumin to creatinine ratio (i.e., >30 mg/g Cr) and/or urine protein to creatinine ratio (i.e., ≥30 mg/g Cr) and/or quantitative 24 hours proteinuria (≥150 mg/day).

### Criteria for assessment of kidney failure etiology

The etiology of kidney failure was assessed using the diagnosis made by nephrologist or another treating physician. If this was unavailable, the criteria described below (based on literature [10,11,12,13,14] and our clinical expertise) were used to determine the probable cause of kidney failure.

1. **Diabetic kidney disease** (DKD) was identified if a patient had an established diagnosis of DM and persistent albuminuria exceeding 300 mg/24 hours (or 200 mg/min), or an albumin-to-creatinine ratio (ACR) greater than 300 mg/g, validated in at least two out of three samples, accompanied by diabetic retinopathy and no indications of other renal disorders.
2. **Hypertensive nephrosclerosis** was identified if any of the following conditions were present: a prolonged history of hypertension preceding kidney dysfunction (at least five years), presence of left ventricular hypertrophy, hypertensive retinopathy, and no evidence of any other CKD etiology.
3. **Chronic glomerulonephritis** was classified based on the following criteria: 1) the pattern of urinary abnormalities, including glomerular hematuria (established by the presence of urinary red blood cell (RBC) casts (of any number) or hematuria in which a significant proportion of RBCs are acanthocytes) or the findings of dysmorphic RBCs in the urine sediment), and/or persistent proteinuria seen on urinalysis, with/without abnormal urinary cast; 2) abnormal serological and/or immunological results; and/or 3) kidney biopsy.
  In primary glomerulonephritis, disease is almost entirely confined to kidney, whereas secondary glomerulonephritis is associated with more diffuse inflammation.
4. **Urolithiasis** was identified when the patient had evidence of solid urinary stones, with or without hematuria and presence of hydronephrosis/hydroureter, as evidenced by any imaging/procedure involving the kidney and urinary tract.
5. **Autosomal dominant polycystic kidney disease (ADPKD)** was identified if the patient had enlarged/palpable kidneys with bilateral complex renal cysts, with/without family history of polycystic kidney disease and a history of flank pain. Imaging evidence of polycystic kidney with/without evidence of cystic disease in another organ.
6. **Toxic nephropathy** is diagnosed when the patient has a strong record exposure to nephrotoxic substances, such as NSAIDS, traditional/herbal medicines, carbonated drinks, etc. in the absence of any other CKD etiology.
7. **Unknown** etiology was considered when no apparent cause could be found.
8. **Other** attributable causes were diagnosed based on kidney imaging/laboratory parameters or kidney biopsies.

### Statistical analysis

Descriptive data will be summarized for continuous variables as mean+standard deviation (SD) for normally distributed data and median (interquartile range [IQR]) for non-normally distributed data. Categorical data will be expressed as proportions. The distribution of PRD was stratified by gender, age group, and KRT modalities. Kidney function was described based on different PRDs and presented in graphs. We analyzed data of “unknown etiology” based on demographic profiles, kidney function and presence of urine abnormalities.

### Missing data

The frequency of missing variables ranged from 3.2% (vascular access) to 60.7% (eGFR at KRT initiation) of the baseline characteristic data. The highest rate of missing data was identified on laboratory parameters, ranging from 58.3% (hemoglobin) to 85.6% (calcium). The analysis was conducted using all available data. All statistical analyses were executed through SPSS for Windows version 25.

## Results

This study included 1,152 kidney failure patients treated with hemodialysis (68.1%), kidney transplantation (24.4%), and CAPD (7.5%). Among 281 kidney transplant patients, 261 (93%) patients had undergone dialysis (251 on HD and 10 on CAPD) prior to transplantation, while 20 (7%) were transplanted before starting dialysis.

### Description of study participants

Our patients were predominantly male (58.5%) with a median (IQR) age of 48 (37–58) years at KRT initiation (Table 1). Javanese (29.5%) and Betawi (22.4%) constituted the predominant ethnic groups represented in this cohort. Hypertension (74.2%) was the primary comorbidity, followed by DM (30.1%) and cardiovascular disease (11.5%). A significant proportion of patients (10.6%) also had hepatitis C. Nearly 15% of patients had a family history of kidney disease and urolithiasis, whereas smoking, chronic NSAID exposure, and exposure to other nephrotoxic agents (i.e., carbonated drinks, taurine, herbal medicines) were reported by 34.8%, 22.4%, and 18.2% of patients, respectively.

**Table 1:** Baseline characteristics of study participants.


VARIABLES		RESULTS

Total patients (n)		1,152

*KRT modalities, n (%)	Hemodialysis	785 (68.1)

	CAPD	86 (7.5)

	Kidney transplant	281 (24.4)

Male, n (%)		674 (58.5)

Age, median (IQR) years		52 (41–61)

Age at KRT presentation, median (IQR) years		48 (37–58)

Ethnicity, n (%)	Javanese	341 (29.5)

	Betawi	258 (22.4)

	Sundanese	132 (11.5)

	Batak	67 (5.8)

	Malay	13 (1.1)

	Balinese	3 (0.3)

	Others	204 (17.7)

	Missing data	134 (11.6)

Comorbidities, n (%)	Hypertension	855 (74.2)

	Diabetes mellitus	347 (30.1)

	Cardiovascular disease	133 (11.5)

	Stroke	44 (3.8)

	Malignancy	21 (1.8)

	Hepatitis B	46 (4.0)

	Hepatitis C	122 (10.6)

	Others	165 (14.3)

Medical history, n (%)	Family history with kidney disease	171 (14.8)

	Smoking	401 (34.8)

	NSAID exposure	258 (22.4)

	History of urolithiasis	172 (14.8)

	Other nephrotoxic exposure	210 (18.2)

^¥^eGFR at KRT initiation, median (IQR) ml/minute/1.73 m^2^		5.90 (4.0–8.34)
	
	<5 ml/minute/1.73 m^2^, n(%)	156 (34.4)

	5–10 ml/minute/1.73 m^2^, n(%)	239 (52.8)

	>10 ml/minute/1.73 m^2^, n(%)	58 (12.8)

	Missing data, n(%)	699 (60.7)

Duration on KRT, median (IQR) years		3.0 (1.0–5.0)

Vascular access for HD, n (%)	AV Fistula	476 (60.6)

	CVC	271 (34.5)

	Others	13 (1.7)

	Missing data	25 (3.2)


* The KRT initiation date was made accordingly to their current KRT modality (i.e., dialysis or kidney transplantation). Therefore, for the kidney transplant group, the time of KRT initiation was refer to the time the subjects received their kidney transplant. ^¥^ Data was collected from 39.3% (n = 453/1152) participants with complete initial laboratory parameters. AV fistula *arteriovenous fistula*; CAPD *continuous ambulatory peritoneal dialysis*; CVC *central venous catheter*; eGFR *estimated glomerular filtration rate*; KRT *kidney replacement therapy*.

The median (IQR) eGFR at KRT initiation was 5.9 (4.0–8.34) ml/minute/1.73 m^2^, and only 12.8% of patients initiated dialysis at eGFR >10 ml/minute/1.73 m^2^. The median (IQR) KRT duration was 3 (1.0–5.0) years. Arteriovenous fistula was the vascular access in 60.6% of HD patients, while 34.5% of participants had a central venous catheter.

During the initiation of KRT, both the median (IQR) for hemoglobin and serum albumin were low (8.90 (7.4–10.1) g/dL and 3.39 (2.9–3.9) mg/dL), respectively, while the phosphate level was high at 5.30 (4.0–7.25) mg/dL (Table 2). Serology revealed that 4.5% (n = 15/332) and 4.9% (n = 16/329) of patients had positive results for hepatitis B and hepatitis C, respectively. In addition, 1.1% (n = 3/284) of patients were HIV positive.

**Table 2:** Laboratory parameters at KRT initiation.


VARIABLES	RESULTS

Hemoglobin (n = 480), median (IQR) g/dl	8.9 (7.4–10.1)

Leucocyte (n = 478), median (IQR) per mm^3^	8,570 (6,592.5–11,255)

Thrombocyte (n = 479), median (IQR) cells/µL	243,000 (186,000–308,000)

Albumin (n = 195), median (IQR) mg/dL	3.39 (2.9–3.9)

Calcium (n = 166), median (IQR) mg/dL	8.0 (7.07–8.92)

Phosphate (n = 157), median (IQR) mg/dL	5.3 (4.0–7.25)

Sodium (n = 434), median (IQR) meq/L	136 (132–140)

Potassium (n = 441), median (IQR) meq/L	4.5 (3.8–5.1)

HBsAg positive (n = 332), n (%)	15 (4.5)

Anti-HCV positive (n = 329), n (%)	16 (4.9)

Anti-HIV positive (n = 284), n (%)	3 (1.1)


HBsAg *hepatitis B surface antigen*; HCV *hepatitis C virus*; KRT *kidney replacement therapy*.

### The etiology of kidney failure

The highest prevalent PRD was DKD (27.2%), followed by glomerulonephritis (13%), hypertension (11.5%), and urolithiasis (10.3%) (Table 3). Within the DKD group, 12.9% of patients had hematuria. Among subjects with glomerulonephritis, 14.7% were diagnosed with secondary glomerulonephritis, with 91% of this subgroup having lupus nephritis. Out of 58 patients with other causes of kidney disease, 37.9% were related to pre-eclampsia, 19% with urinary tract and gynecological malignancy, 12.1% had CAKUT (congenital anomalies on kidney and urinary tract) as well as cardiorenal syndrome, and 6.9% presented with infection-related kidney disease (HIV and hepatitis C-associated).

**Table 3:** Etiology of kidney disease in the study population.


ETIOLOGY OF KIDNEY DISEASE	N (%)

Diabetic kidney disease	313 (27.2)

Hypertensive nephrosclerosis	132 (11.5)

Glomerulonephritis	150 (13.0)

Primary glomerulonephritis	128 (85.3)

Secondary glomerulonephritis	22 (14.7)

Lupus nephritis	20 (91)

Others	2 (9)

Urolithiasis	119 (10.3)

Autosomal dominant polycystic kidney disease	17 (1.5)

Toxic Nephropathy	5 (0.4)

Others:	58 (5.0)

CAKUT	7 (12.1)

Urinary tract and gynecology malignancy	11 (19.0)

Cardiorenal syndrome	7 (12.1)

Pre-eclampsia	22 (37.9)

Infection^*^	4 (6.9)

Miscellaneous	7 (12.1)

Unknown	358 (31.1)


* Infection (3 subjects: related to HIV-associated nephropathy, 1 subject: related to hepatitis C infection).

No clear cause of kidney failure could be determined in 31.1% of patients. This group had the highest distribution in Balinese and others ethnic groups (66.7% and 34.1%, respectively), with a mean age of 56.53 ± 12.88 years, and a median (IQR) eGFR was 4.4 (3.0–7.0) ml/minute/1.73 m^2^. Furthermore, hematuria was observed in 10.6% of patients, as well as albuminuria (microalbuminuria 26.3% and macroalbuminuria 1.2%) (Supplementary Table S1).

**Figure 1:**
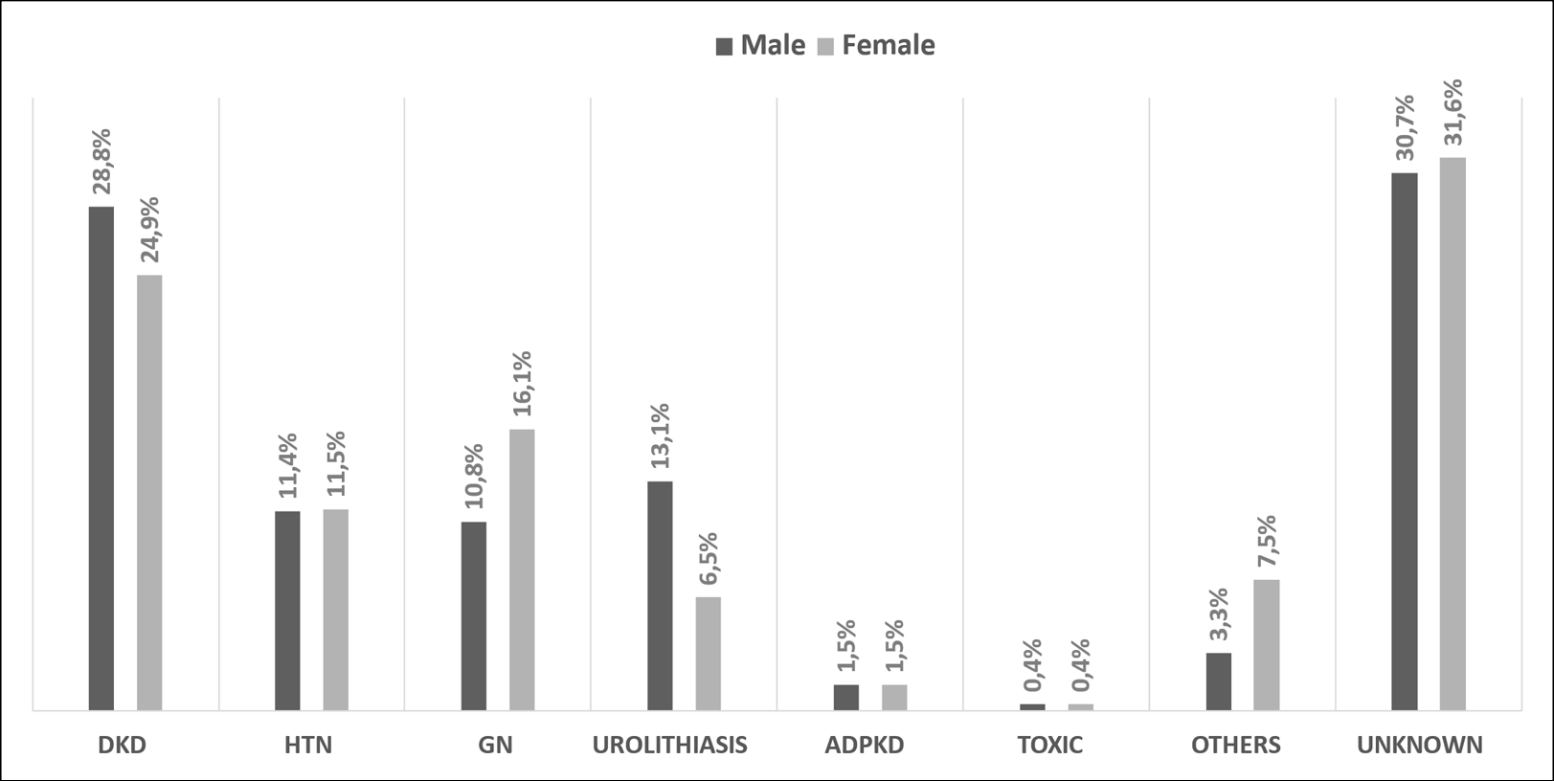
The distribution of ESKD etiology stratified by gender. *Notes*: DKD = diabetic kidney disease; HTN = hypertensive nephrosclerosis; GN = glomerulonephritis; ADPKD = autosomal dominant polycystic kidney disease; Toxic = toxic nephropathy.

Subsequently, we assessed whether male and female patients were equally distributed within the etiology of kidney failure, i.e., hypertensive nephrosclerosis (11.4%vs11.5%), ADPKD (1.5%vs1.5%), and toxic nephropathy (0.4%vs0.4%). The occurrence of DKD was higher in males than females (28.8% vs 24.9%), along with urolithiasis (13.1% vs 6.5%) (Figure 1).

**Figure 2:**
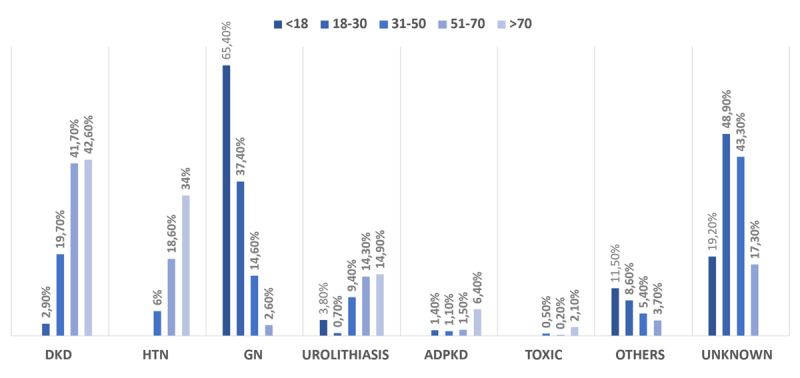
The distribution of ESKD etiology stratified by age groups. *Notes*: DKD = diabetic kidney disease; HTN = hypertensive nephrosclerosis; GN = glomerulonephritis; ADPKD = autosomal dominant polycystic kidney disease; Toxic = toxic nephropathy.

In Figure 2, it is evident that glomerulonephritis was predominantly found in younger patients (median (IQR): 32 (24–40) years) as opposed to DKD (mean 57.9 + 10.1 years). Hypertensive nephrosclerosis and urolithiasis were primarily observed in the older age group (mean age 61.6 + 9.0 and 44.9 + 11.6 years, respectively). The distribution of unknown causes was mainly among the age group of 30–69 years (mean age 56.5 + 12.9 years).

**Figure 3:**
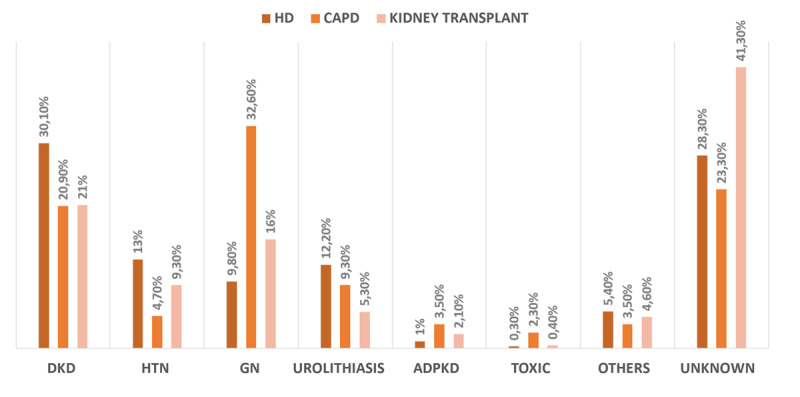
The distribution of ESKD etiology stratified by KRT modalities. *Notes*: DKD = diabetic kidney disease; HTN = hypertensive nephrosclerosis; GN = glomerulonephritis; ADPKD = autosomal dominant polycystic kidney disease; Toxic = toxic nephropathy.

The greatest proportion of DKD patients were assigned to hemodialysis (30.1%) while glomerulonephritis patients were likely to be treated with CAPD. While the largest prevalent of kidney transplant patients had unknown cause (41.3%), followed by DKD (21%) (Figure 3).

The median (IQR) eGFR to start KRT was highest in DKD, subsequently ADPKD and hypertensive nephropathy (7.27 (5–9.52); 6.25 (4.2–7.93), and 6.0 (4.05–9.0) ml/minute/1.73 m^2^; respectively) (Supplementary Table S2).

## Discussion

In the present study, we aimed to describe the etiology of kidney failure in Jakarta. Diabetic kidney disease, glomerulonephritis, hypertensive nephropathy, and urolithiasis appeared to be the prevalent causes of kidney failure. The majority of our patients were male, with a median age of 48 years, and hemodialysis was the preferred choice of KRT.

The patients in this cohort presented with kidney failure at a comparatively younger age than what has been reported in earlier studies from Europe, China, and the US [15,16,17]. For instance, in the US, the adjusted prevalence of ESKD was higher among the elderly, with 7,473 cases per million population (pmp) among those aged ≥75 years and 7,419 cases pmp among those aged 65–74 years in 2019 [15]. Similarly, in Europe, the incidence of KRT was found to be highest among the elderly (75–84 year), and in China, the average age of chronic hemodialysis patients was 58.7 ± 3.5 years [16,17]. There are several reasons for this difference. First, it might reflect an inability to identify kidney dysfunction in the early phases because of the unawareness of kidney disease or due to inadequate screening and diagnostic tools at this stage. We observed that a substantial proportion of patients had risk factors for CKD (i.e., family history of kidney disease or exposure to nephrotoxic agents), but most of them were not screened for kidney injury in the early stage. Early identification of the at-risk population may help slowing the progression of CKD. The second explanation for the younger age of kidney failure patients in Indonesia is linked to the higher risk of death among elderly CKD patients compared to younger age groups. In other words, elderly patients have a higher risk of dying before progression towards kidney failure develops [18,19,20,21]; however, there is no data available on this trend in Indonesia. Third, younger patients may have better access to KRT than older patients, whereas in the latter group conservative treatment is chosen more frequently [18,22].

The eGFR at time of dialysis initiation varies across studies. In general, the mean eGFR for starting dialysis in Asia is lower when compared to that of the US or Europe [23,24,25,26,27]. Our finding suggested that the median eGFR to start dialysis was 5.9 ml/minute/1.73 m^2^, although 12.8% of patients commenced dialysis with an eGFR >10 ml/minute/1.73 m^2^. In line with other observational studies [23,26,27,28], patients with DKD tended to start dialysis earlier. Another recent study reported a high rate of crash-landers (83%) where 67% of patients did not receive any prior treatment for their kidney dysfunction [4]. This implies the need for the urgent identification of high-risk CKD subjects. Previous studies have suggested that patients who start with hemodialysis urgently have a six-fold higher three-month mortality rate than those who began with planned hemodialysis [29]. In daily practice, it is often challenging to identify the cause of CKD when patients present at the stage of kidney failure, as the extensive, non-specific glomerulosclerosis in renal biopsy precludes the identification of the PRD. When patients encounter kidney failure, the transition to KRT is likely to be the only option.

This study showed that DKD is the most common cause of kidney failure. Compared to a previous report in 2000, there has been a shift from glomerulonephritis to DKD as the leading cause of kidney failure [30]. This shift is in line with the phenomenon of epidemiological transition occurring in Indonesia [8–9]. However, since our study used data from patients residing in urban areas, our observations may not be generalizable to all regions of Indonesia, particularly for patients living in rural and remote areas. Although most DKD patients present with kidney failure at older age, we also observed a group of patients presenting before the age of 30 years. These findings are consistent with other reports showing that the epidemiology of type 2 DM is evolving from a chronic disease affecting the elderly to one that is prevalent in younger patients as well [31,32,33]. Apart from albuminuria, which is the primary clinical manifestation of DKD, we discovered that 13% of our patients had hematuria. This may be due to a different clinical disease course compared to the non-hematuria group [34], or it may be due to another PRD since the DKD diagnosis was not biopsy-proven. Ninety million people in South-East Asia living with DM, and it is anticipated that the greatest prevalence rise will occur in countries with middle-income [33]. Moreover, nearly half of type 2 DM patients will develop DKD. In view of this high risk of progressive DKD, there is an urgent need to establish appropriate screening program for this specific population in order to prevent kidney complications and kidney failure development.

Lupus nephritis was the most common cause of secondary glomerulonephritis, and the rate is significantly higher than previous reports [30,35,36,37]. Lupus patients may be underdiagnosed due to a lack of diagnostic facilities and limited awareness and knowledge of the disease. For instance, not all hospitals in Indonesia have access to all immunological tests for lupus, and kidney biopsy services are only available in a small number of type A hospitals. And to date, we do not have electron microscope facilities to support the diagnosis of kidney disorders in Indonesia. Moreover, previous biopsy study on lupus nephritis showed a high prevalence of proliferative lesions (74.8%), which could contribute to a worse kidney prognosis, including kidney failure [37]. Although various medications are available for lupus nephritis treatment in Indonesia [38,39], treatment options are still suboptimal due to limited national insurance coverage and reimbursement exclusions [39].

Another remarkable observation in our study is the higher prevalence of urolithiasis in our cohort (10% of patients) compared to reports from western countries (0.2%–5.62%) [40,41]. In Asia, the prevalence of urolithiasis in general population ranges from 1–19% [42], although evidence of kidney failure related to this condition is limited. Factors contributing to urolithiasis development in Asia include dietary habits (such as a Western diet and lower fluid intake), warm climate (hot temperatures and longer sunshine exposure), and the increasing prevalence of metabolic syndrome [42]. Access to sufficient clean water may also play a role in kidney stone development, as 36.4% of our population lacks access to clean water, as indicated by our previous study [9]. The growing incidence of metabolic diseases, such as obesity and diabetes, each affecting almost a third of our study population, has an impact on stone development.

The PRD was unable to be established in 31.1% of patients. Determining the underlying etiology is challenging since most patients presented at the terminal stage and no previous clinical data were recorded. Moreover, histopathological confirmation was made in less than one percent of patients. We may argue that the inability to accurately define PRD is primarily due to the inability to detect high-risk population early or the combined effect of environmental and lifestyle exposures which are associated to the non-traditional CKD etiology (CKD of unknown etiology/CKDu). Risk factors include heat stress, dehydration, agrochemical, heavy metals, water sources, locally made alcohol beverages, snake bites, infections and a family history of CKD [43]. A recent study of rice farmers in West Java found a CKDu prevalence of 18.6%, which increased with longer insecticide use [44]. Even though the likelihood of CKDu occurrence in our study group is low, we should not overlook this alternative diagnosis and additional study may be necessary. Our study was unable to fully examine CKD risk factors, although we found that a family history of kidney disease, smoking, nephrotoxic exposure, history of urolithiasis, and viral hepatitis were relatively high. Further study is needed to clearly examine the relationship between these risk factors and CKD development in our population.

Our study had several strengths and limitations. This is currently the most extensive study analyzing the etiology of kidney failure in Indonesia. Our findings are significant as they provide crucial information on the present epidemiology of kidney failure in Indonesia, and may affect policies and strategies on kidney disease management. However, there are limitations to our study. Firstly, it was carried out in five tertiary-care centers located only in urban areas on Java Island (specifically Jakarta). As a result, our findings may not accurately reflect the causes of kidney failure across Indonesia, particularly in rural and remote regions. Secondly, due to the preponderance of the Javanese and Betawi ethnic groups, our results cannot be generalized to the entire Indonesian population. Finally, due to the retrospective nature of this study, we were unable to retrieve the complete set of clinical data for some patients. For instance, the kidney biopsy examination was accessible to a mere 0.4% of patients, while the initial laboratory parameter at the KRT initiation was unavailable for as many as 86% of patients unless the imputation strategy was employed in the statistical analysis. The proportion of the PRD diagnosis extracted from the medical record was about 52.4%. Nonetheless, it was not possible to distinguish whether the diagnosis was made by the nephrologist or any other treating physician. Nevertheless, the author meticulously examined the clinical data of all patients and incorporated the information to enhance the PRD diagnosis according to the study criteria.

This study will guide us toward a list of potential issues that require to be addressed in a subsequent study among Indonesian population, including 1) the need for early identification on individuals at-risk for CKD; 2) the importance of population education on kidney health and CKD-related risk factors to raise awareness of kidney diseases, as well as to seek for medical advice earlier for their potential kidney problems; 3) the need for further improvement of our health referral system for primary or secondary CKD prevention and to acquire a proper nephrologist care; and 4) to provide optimum diagnostic tools to identify and treat kidney involvement in high risk CKD patients.

## Conclusion

Our study suggests that DKD is the major cause of kidney failure, followed by glomerulonephritis, hypertensive nephrosclerosis, and urolithiasis among tertiary-care hospitals in Jakarta, Indonesia. This study emphasizes the need for a better approach to primary prevention of DM and for better recognition of glomerulonephritis at an early stage, as both activities might significantly reduce the rate of kidney failure in Indonesia.

## Additional File

The additional file for this article can be found as follows:

10.5334/aogh.4071.s1 Supplementary Tables. Table S1 and S2.
