# Supplementary material for: The Etiology of Kidney Failure in Indonesia: A Multicenter Study in Tertiary-Care Centers in Jakarta
Source: Ann Glob Health. 2023 Jun 1;89(1):36. doi: 10.5334/aogh.4071 (PMC10237240; doi:10.5334/aogh.4071)
Supplement: Supplementary Tables. — Table S1 and S2. [file agh-89-1-4071-s1.pdf]

Supplementary Materials

Supplementary Table S1. Characteristic of subjects with unknown etiology of KF

**Variables**		**Results**
Male, n (%)		207 (30.7)
Age at ESKD presentation, mean±SD years		56.53 ± 12.88
Ethnicity	Javanese	96 (28.2)
	Betawi	84 (32.6)
	Sundanese	58 (28.4)
	Batak	20 (29.9)
	Malay	4 (30.8)
	Balinese	2 (66.7)
	Others	45 (34.1)
eGFR, median (IQR) ml/min/1.73m^2		4.4 (3.0–7.0)
Albuminuria, n (%)	Microalbuminuria	88 (26.3)
	Macroalbuminuria	4 (1.2)
Hematuria, n (%)		36 (10.6)

Supplementary Table S2. The distribution of eGFR at the initiation of KRT based on kidney failure etiology

**Etiology of Kidney Failure**	**eGFR (ml/minute/1.73 m^2)** [¥]
Diabetic kidney disease, median (IQR)	7.27 (5.00 – 9.52)
Hypertensive nephrosclerosis, median (IQR)	6.00 (4.05 – 9.00)
Glomerulonephritis, median (IQR)	5.00 (3.08 – 6.25)
Urolithiasis, median (IQR)	5.98 (3.00 – 8.00)
Autosomal dominant polycystic kidney disease, median (IQR)	6.25 (4.20 – 7.93)
Toxic nephropathy[¶]	10.60
Others, median (IQR)	5.00 (4.00 – 7.70)
Unknown, median (IQR)	4.40 (3.00 – 7.00)
Missing data, n (%)	699 (60.7)

[¶]Data available from a single patient only

eGFR *estimated glomerular filtration rate*; KRT *kidney replacement therapy*
